# Methylation is maintained specifically at imprinting control regions but not other DMRs associated with imprinted genes in mice bearing a mutation in the *Dnmt1* intrinsically disordered domain

**DOI:** 10.3389/fcell.2023.1192789

**Published:** 2023-08-04

**Authors:** Shaili Regmi, Lana Giha, Ahado Ali, Christine Siebels-Lindquist, Tamara L. Davis

**Affiliations:** Department of Biology, Bryn Mawr College, Bryn Mawr, PA, United States

**Keywords:** genomic imprinting, DNA methylation, DMNT1, epigenetics, differentially methylated regions

## Abstract

Differential methylation of imprinting control regions in mammals is essential for distinguishing the parental alleles from each other and regulating their expression accordingly. To ensure parent of origin-specific expression of imprinted genes and thereby normal developmental progression, the differentially methylated states that are inherited at fertilization must be stably maintained by DNA methyltransferase 1 throughout subsequent somatic cell division. Further epigenetic modifications, such as the acquisition of secondary regions of differential methylation, are dependent on the methylation status of imprinting control regions and are important for achieving the monoallelic expression of imprinted genes, but little is known about how imprinting control regions direct the acquisition and maintenance of methylation at these secondary sites. Recent analysis has identified mutations that reduce DNA methyltransferase 1 fidelity at some genomic sequences but not at others, suggesting that it may function differently at different loci. We examined the impact of the mutant DNA methyltransferase 1 P allele on methylation at imprinting control regions as well as at secondary differentially methylated regions and non-imprinted sequences. We found that while the P allele results in a major reduction in DNA methylation levels across the mouse genome, methylation is specifically maintained at imprinting control regions but not at their corresponding secondary DMRs. This result suggests that DNA methyltransferase 1 may work differently at imprinting control regions or that there is an alternate mechanism for maintaining methylation at these critical regulatory regions and that maintenance of methylation at secondary DMRs is not solely dependent on the methylation status of the ICR.

## 1 Introduction

Genomic imprinting results in parent of origin-specific monoallelic expression of approximately 150 genes in mammals (Morison et al., 2005; https://www.geneimprint.com/site/genes-by-species.Mus+musculus). Parent of origin-specific DNA methylation at imprinting control regions (ICRs) is established during gametogenesis, inherited at fertilization, maintained throughout development, and serves as the primary imprinting mark; as such, it is responsible for distinguishing the parental alleles from each other and regulating their expression accordingly (Barlow and Bartolomei, 2014). Differential methylation of ICRs is therefore essential for establishing imprints, and recent studies have further proven the importance of maintaining differential methylation at ICRs in order to retain monoallelic imprinted expression patterns. Epigenetic editing resulting in methylation of the typically unmethylated maternal tandem repeats within the *Dlk1-Dio3* IG-DMR led to paternalization of the maternal allele, including the acquisition of methylation across the IG-DMR and concomitant silencing of *Meg3* (Kojima et al., 2022). Conversely, targeting TET1 activity to the tandem repeats with the IG-DMR on the typically methylated paternal IG-DMR maternalized the paternal allele as evidenced by decreased methylation across this locus and expression of *Meg3* from the typically silent paternal allele (Kojima et al., 2022).

In addition to the primary, or gametic, differentially methylated regions (DMRs) that function as ICRs and are essential for regulating imprinted expression, some imprinted genes also acquire distinct regions of differential methylation during post-implantation development (Tremblay et al., 1997; Hanel and Wevrick, 2001; Takada et al., 2002; Bhogal et al., 2004; Gagne et al., 2014; Guntrum et al., 2017). Acquisition of parent of origin-specific methylation at these secondary, or somatic, DMRs is dependent on the epigenetic state of the corresponding primary DMR, although the exact mechanisms driving methylation acquisition at secondary DMRs are not well understood (Saito et al., 2018; Hara et al., 2019). For example, epigenetic alteration of methylation at the IG-DMR or targeted deletion of IG-DMR sequences directly influences the methylation state of the corresponding secondary DMR located at the *Gtl2* (*Meg3*) promoter (Aronson et al., 2021; Kojima et al., 2022), highlighting the relationship between these two elements. While differential methylation of the primary DMRs is essential for establishing the parent of origin epigenotype at each imprinting cluster, the subsequent acquisition of parent of origin-specific methylation at secondary DMRs appears to be important for maintaining parent of origin-specific expression of individual loci (Stöger et al., 1993; Constância et al., 2000; Bhogal et al., 2004; Kobayashi et al., 2009; Kagami et al., 2010; John and Lefebvre, 2011; Nakagaki et al., 2014; Aronson et al., 2021; Kojima et al., 2022).

Despite the demonstrated importance of differential methylation at secondary DMRs in the regulation of the individual imprinted genes with which they are associated, DNA methylation is less consistent at secondary DMRs than at primary DMRs (Tremblay et al., 1997; Hanel and Wevrick, 2001; Takada et al., 2002; Yatsuki et al., 2002; Arnaud et al., 2003; Coombes et al., 2003; Ono et al., 2003; Nowak et al., 2011; Woodfine et al., 2011; Arand et al., 2012; Gagne et al., 2014; Guntrum et al., 2017; Nechin et al., 2019). Investigation of DNA methylation patterns at secondary DMRs revealed high levels of methylation asymmetry (Guntrum et al., 2017; Nechin et al., 2019), which may be a result of TET activity at these loci which would lead to 5-hydroxymethylcytosine enrichment and subsequent active or passive demethylation (Valinluck and Sowers, 2007; Tahiliani et al., 2009; He et al., 2011; Ito et al., 2011; Kohli and Zhang, 2013). Despite the high levels of methylation asymmetry observed at secondary DMRs, overall levels of DNA methylation remain consistent across development, consistent with the hypothesis that the epigenetic profile at primary DMRs directs methylation acquisition at secondary DMRs throughout development (Bhogal et al., 2004; Gagne et al., 2014; Guntrum et al., 2017; Nechin et al., 2019).

The establishment and maintenance of DNA methylation is achieved by DNA methyltransferases (Dnmts). Dnmt3a and Dnmt3b function as *de novo* methyltransferases while Dnmt1 functions as the maintenance methyltransferase (Li and Zhang, 2014). Dnmt1 plays a critical role in maintaining global methylation, and complete loss of *Dnmt1* activity is embryonic lethal (Li et al., 1992). Dnmt1 has also been shown to be responsible for maintaining methylation at primary DMRs associated with imprinted genes, including during the genome-wide demethylation that occurs during pre-implantation development (Howell et al., 2001; Hirasawa et al., 2008; Liu et al., 2022). Mutation of *Dnmt1* supports the hypothesis that it may function differently at different genomic locations. Dissection of *Dnmt1* via mutational analysis has identified specific regions of the Dnmt1 protein that are essential for maintaining non-imprinted but not imprinted methylation patterns and *vice versa* (Borowczyk et al., 2009; Shaffer et al., 2015). These mutations are located in the intrinsically disordered domain (IDD) of Dnmt1, suggesting that different sequences within this region may influence Dnmt1 activity at different targets within the mouse genome (Shaffer et al., 2015).

Herein, we describe our investigation of the *Dnmt1* P allele. The P allele is a mutation in the mouse *Dnmt1* IDD that replaces six codons with the corresponding rat sequence (Shaffer et al., 2015). Work by Shaffer and others (Shaffer et al., 2015) illustrated that *Dnmt1* ^*P/P*^ is lethal, likely due to a dramatic reduction in global DNA methylation. Despite the overall reduction in DNA methylation globally and at IAP sequences, methylation was relatively well maintained at primary DMRs associated with imprinted loci (Shaffer et al., 2015). We compared DNA methylation levels in *Dnmt1* ^*P/P*^ mutant embryos across development to determine whether methylation is also maintained better at secondary DMRs, whose methylation status is dependent on the methylation state of the corresponding primary DMR. Our results illustrate that methylation at secondary DMRs associated with imprinted genes is dramatically reduced in *Dnmt1* ^*P/P*^ mutants, supporting the hypothesis that methylation is maintained differently at different sequences and that different factors may be responsible for maintaining methylation at primary vs. secondary DMRs.

## 2 Materials and methods

### 2.1 Mice

Sv/129 mice heterozygous for the *Dnmt1* P allele mutation (Shaffer et al., 2015) were obtained from Dr. Mellissa Mann (Magee-Womens Research Institute, Pittsburg, PA). Natural matings between heterozygous pairs were used to generate *Dnmt1* ^*+/+*^, *Dnmt1* ^*P/+*^ and *Dnmt1* ^*P/P*^ embryos, which were collected at 9.5, 12.5, 15.5 and 18.5 days post coitum (dpc). Natural matings were also used to generate offspring in order to maintain the *Dnmt1* P allele in the colony. Ethical approval for procedures involving animals was granted by the Bryn Mawr College Institutional Animal Care and Use Committee, PHS Welfare Assurance Number A3920-01.

### 2.2 Genotyping

Genotypes were determined using a PCR-based assay described by Shaffer *et al.* (Shaffer et al., 2015). Briefly, DNA was extracted from embryo or 3–4 weeks mouse tails using proteinase K digestion and genomic DNA was purified using a Genomic DNA Clean & Concentrator kit (Zymo Research, Irvine, CA, cat#D4011). PCR using oligonucleotides flanking the P allele mutation was followed by restriction digestion with *Ava*I, and wild type vs. mutant P alleles were distinguished by agarose gel electrophoresis (wild type allele, 627 bp; P allele, 447 + 180 bp). Chi-square goodness of fit tests were conducted in Microsoft Excel, using the raw number of *Dnmt1* ^*+/+*^, *Dnmt1* ^*P/+*^ and *Dnmt1* ^*P/P*^ embryos or pups collected at each developmental stage, to determine whether the observed values deviated significantly from the Mendelian ratios expected from crosses between heterozygous pairs.

### 2.3 DNA purification, template preparation and bisulfite sequence analysis

Genomic DNA was isolated from 9.5, 12.5, 15.5 and 18.5 dpc embryo heads following proteinase K digestion and a series of phenol/chloroform extractions as described previously (Davis et al., 1999). Purified DNA was subjected to bisulfite mutagenesis using an EZ DNA Methylation-Direct kit (Zymo Research, Irvine, CA, cat#D5020). Mutagenized DNA was subjected to nested or semi-nested PCR amplification; primers, PCR annealing temperatures and expected second round PCR product size for each locus analyzed are detailed in Supplementary Table S1. Resulting amplicons were purified from agarose gels using a Zymoclean Gel DNA Recovery kit (Zymo Research, Irvine, CA, cat#D4002) and quantified using a Qubit 3.0 Fluorometer (ThermoFisher Scientific, cat#Q33216). Equimolar amounts of PCR product from multiple loci were combined with a minimum of 50 ng from each amplicon and submitted to Azenta (South Plainfield, NJ) for NextGeneration-based amplicon sequencing. Sequence reads were uploaded to a Galaxy Instance hosted at Bryn Mawr College, paired and processed using fastp, mapped to known target sequences, and analyzed for non-CpG bisulfite conversion efficiency as well as for the presence of cytosines vs. thymines in a CpG context (Afgan et al., 2018). The bisulfite conversion efficiency was >99% for all datasets used in this analysis.

### 2.4 Analysis of downsampled sequences

Downsampling of NGS data was performed using Galaxy tools to obtain 20–25 sequencing reads for each locus analyzed. Percent methylation for each strand was calculated and the raw data from each allele in *Dnmt1* ^*+/+*^ and *Dnmt1* ^*P/P*^ embryos was ranked and assessed for statistically significant differences using a Mann-Whitney U test (http://vassarstats.net/utest.html).

## 3 Results

### 3.1 The *Dnmt1* P allele alters sequences uniquely present in *Mus* and *Rattus*

**FIGURE 1:**
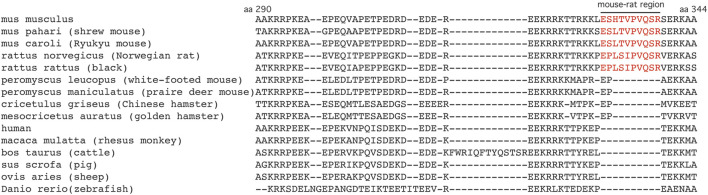
Alignment of Dnmt1 amino acid sequences 290–344. An 8–10 amino acid sequence (red) within the Dnmt1 intrinsically disordered domain is present in *Mus musculus, Mus pahari, Mus caroli, Rattus norvegicus* and *Rattus rattus*, but is not found in other rodents or non-rodent species. Sequences were aligned using the COBALT multiple alignment tool (https://www.ncbi.nlm.nih.gov/tools/cobalt/cobalt.cgi).

The amino terminal intrinsically disordered domain of Dnmt1, residues 92–391, includes a 160 amino acid region unique to eutherian mammals proposed to play a role in mammalian-specific methylation processes such as genomic imprinting (Borowczyk et al., 2009; Liu et al., 2017). Previous research indicated that different portions of this domain may influence the catalytic activity of Dnmt1 at different sequences (Borowczyk et al., 2009). While the primary sequence of Dnmt1 is highly conserved across species (Supplementary Figures S1A–C), Shaffer *et al.* (Shaffer et al., 2015) identified a 10 amino acid region present in mouse and rat that is not present in humans and suggested that this region may be responsible for species specific methylation. Further analysis illustrated that the mouse-rat region is specific to *Mus* and *Rattus* genera, as it is not present in other rodents, including the closely related deer mouse (*Peromyscus leucopus* and *Peromyscus maniculatus*) (Figure 1; Supplementary Figures S1D). The *Dnmt1* P allele, which substituted the mouse codons specifying LESHTV for the rat codons specifying PEPLSI, is embryonic lethal, displaying dramatically reduced levels of global DNA methylation, indicating that this region does not function similarly in *Mus* vs. *Rattus* (Shaffer et al., 2015).

### 3.2 The *Dnmt1* P allele is neonatal lethal

Embryos homozygous for the *Dnmt1* P allele have dramatically reduced levels of global methylation but methylation was observed to be better maintained at primary DMRs associated with imprinted genes (Shaffer et al., 2015). Since the methylation at secondary DMRs associated with imprinted genes is dependent on the methylation of the corresponding primary DMR (Saito et al., 2018; Hara et al., 2019), we wanted to determine whether *Dnmt1* ^*P/P*^ mutant embryos would also retain most of their methylation at secondary DMRs as a consequence of the methylation profile at the associated primary DMR or whether the preferential retention of methylation at primary DMRs is unique in *Dnmt1* ^*P/P*^ mutant embryos, suggesting either that Dnmt1 functions differently at these sequences or that the maintenance of methylation at primary DMRs can be achieved with other DNA methyltransferases.

We collected and genotyped embryos derived from natural matings between *Dnmt1* ^*P/+*^ mice at 9.5, 12.5, 15.5 and 18.5 dpc; at least four litters were collected at each developmental stage. While there was some deviation from the expected 1:2:1 Mendelian ratio at each developmental stage, none of the differences were significant (Table 1). Furthermore, we did not observe consistent differences in morphology between wild-type, heterozygous or homozygous mutant embryos, suggesting that *Dnmt1* ^*P/P*^ embryos survive throughout gestation (Supplementary Figure S2). In contrast, no *Dnmt1* ^*P/P*^ pups survived beyond 1 day after birth. Three of the 245 pups that were observed following natural matings between *Dnmt1* ^*P/+*^ mice were *Dnmt1* ^*P/P*^, and all three were deceased on postpartum day 1. Of the 242 pups that survived beyond postpartum day 1, all survived into adulthood: 84 of the surviving offspring were wild-type, and 158 were heterozygous for the *Dnmt1* P allele mutation (Table 2). These data indicate that the *Dnmt1* P allele is a neonatal lethal. Our observations of the three dead *Dnmt1* ^*P/P*^ pups indicated that none of them had milk in their stomachs. We hypothesize that *Dnmt1* ^*P/P*^ embryos may survive gestation but are unable to eat and/or breathe after birth and are therefore inviable, and that the majority of the *Dnmt1* ^*P/P*^ pups that were born were consumed by their parents before they were observed on postpartum day 0 or day 1.

**TABLE 1:** Genotypes of embryos derived from *Dnmt1* ^*P/+*^ *x Dnmt1* ^*P/+*^ matings. *p* values were calculated using a chi-square goodness of fit test based on the 1:2:1 Mendelian genotype ratio expected from heterozygous parents; no significant differences from the 1:2:1 predicted ratio were detected.

Embryonic stage	+/+	P/+	P/P	*p*-Value
9.5 dpc	8	13	15	0.0639
12.5 dpc	9	14	8	0.8374
15.5 dpc	6	19	8	0.6065
18.5 dpc	15	26	6	0.1368
	38	72	37	0.9633

**TABLE 2:** Genotypes of viable pups derived from *Dnmt1* ^*P/+*^ x *Dnmt1* ^*P/+*^ matings. *p* values were calculated using a chi-square goodness of fit test based on the 1:2:1 Mendelian genotype ratio expected from heterozygous parents vs. the expected 1:2 ratio for a recessive lethal.

Days post-partum	+/+	P/+	P/P	*p*-Value (1:2:1)	*p*-Value (1:2)
P21-28	84	158	0	2.65 × 10^−18^	0.6494

### 3.3 DNA methylation levels are relatively well maintained at primary DMRs associated with imprinted genes in *Dnmt1* ^*P/P*^ mutant embryos as compared to secondary DMRs and non-imprinted sequences

We analyzed methylation levels at primary and secondary DMRs associated with imprinted loci as well as at non-imprinted loci in DNA derived from 9.5, 12.5, 15.5 and 18.5 dpc wild-type and *Dnmt1* ^*P/P*^ siblings (Table 3). Purified genomic embryo DNA was subjected to bisulfite mutagenesis and target loci were amplified by PCR (Supplementary Table S1). Purified amplicons were quantified and pooled at equimolar amounts prior to Next-Generation sequencing. NGS data was analyzed using a Galaxy instance to determine bisulfite mutagenesis efficiency based on non-CpG cytosine conversion to uracil (thymine) and the frequency of cytosine methylation at CpG dinucleotides. Data were obtained from one wild-type and one *Dnmt1* ^*P/P*^ embryo at 9.5, 12.5 and 15.5 dpc, and from two wild-type and two *Dnmt1* ^*P/P*^ embryos at 18.5 dpc.

**TABLE 3:** Primary and secondary DMRs analyzed within different imprinting clusters.

Imprinting cluster	Primary DMR	Secondary DMR(s)
*Igf2*	*H19* ICR	*H19*-pp (promoter proximal)
*Dlk1*	IG-DMR	*Gtl2*, *Dlk1*
	*Rasgrf1*	
*Pws*	*Snrpn*	*Peg12, Ndn, Magel2, Mkrn3*
*Igf2r*	*Airn*	*Igf2r*
*Kcnq1*	*Kcnq1ot1*	*Cdkn1c*

**FIGURE 2:**
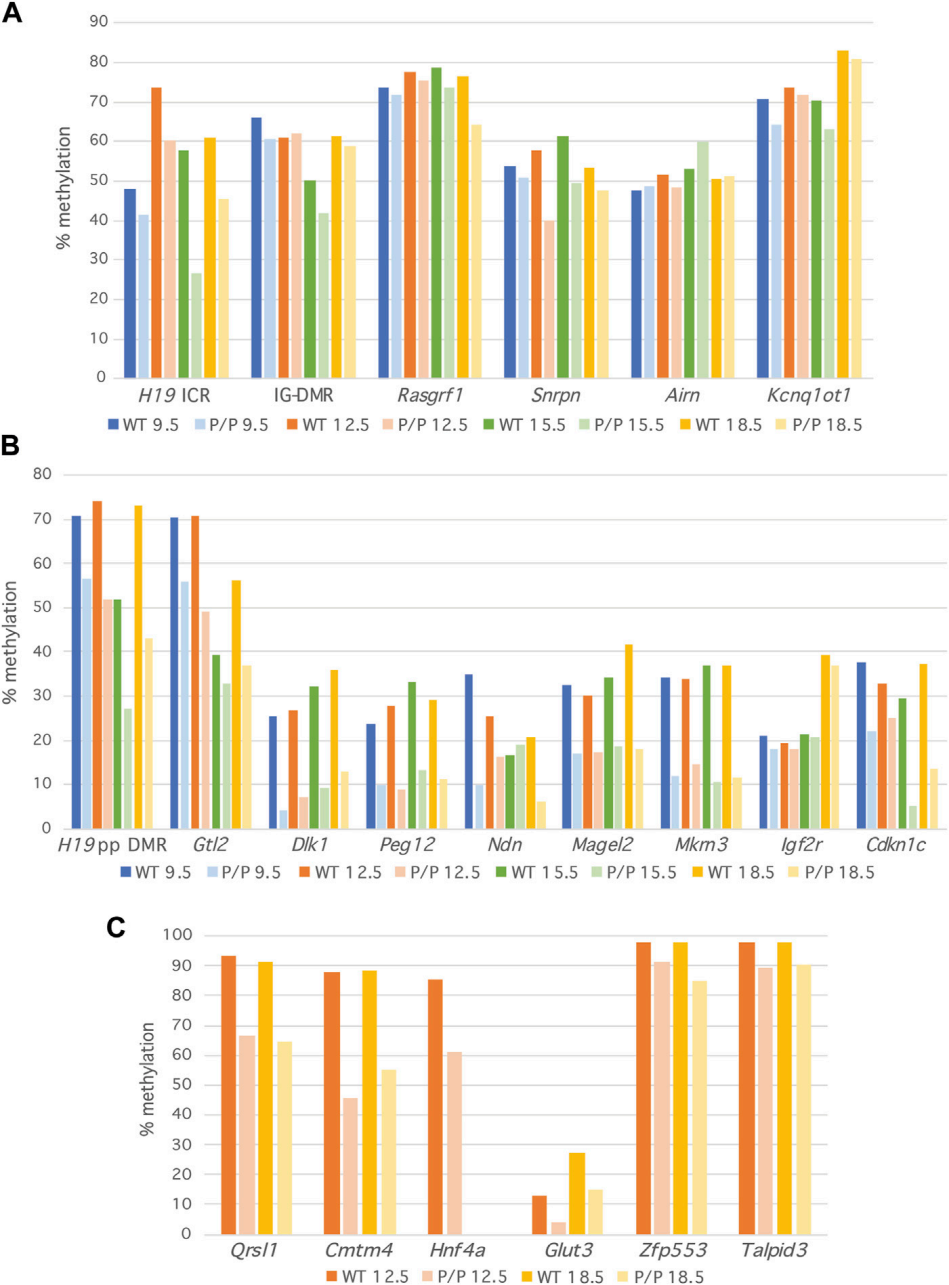
Methylation levels are minimally reduced at primary DMRs in *Dnmt1* ^*P/P*^ (P/P) embryos as compared to their wild-type siblings. Percent methylation derived at each locus from NGS data; 9.5 dpc (blue), 12.5 dpc (orange), 15.5 dpc (green) and 18.5 dpc (yellow). Data were obtained from a single wild-type or *Dnmt1* ^*P/P*^ mutant embryo at each developmental stage. **(A)** Primary DMRs. **(B)** Secondary DMRs. **(C)** Non-imprinted loci.

**FIGURE 3:**
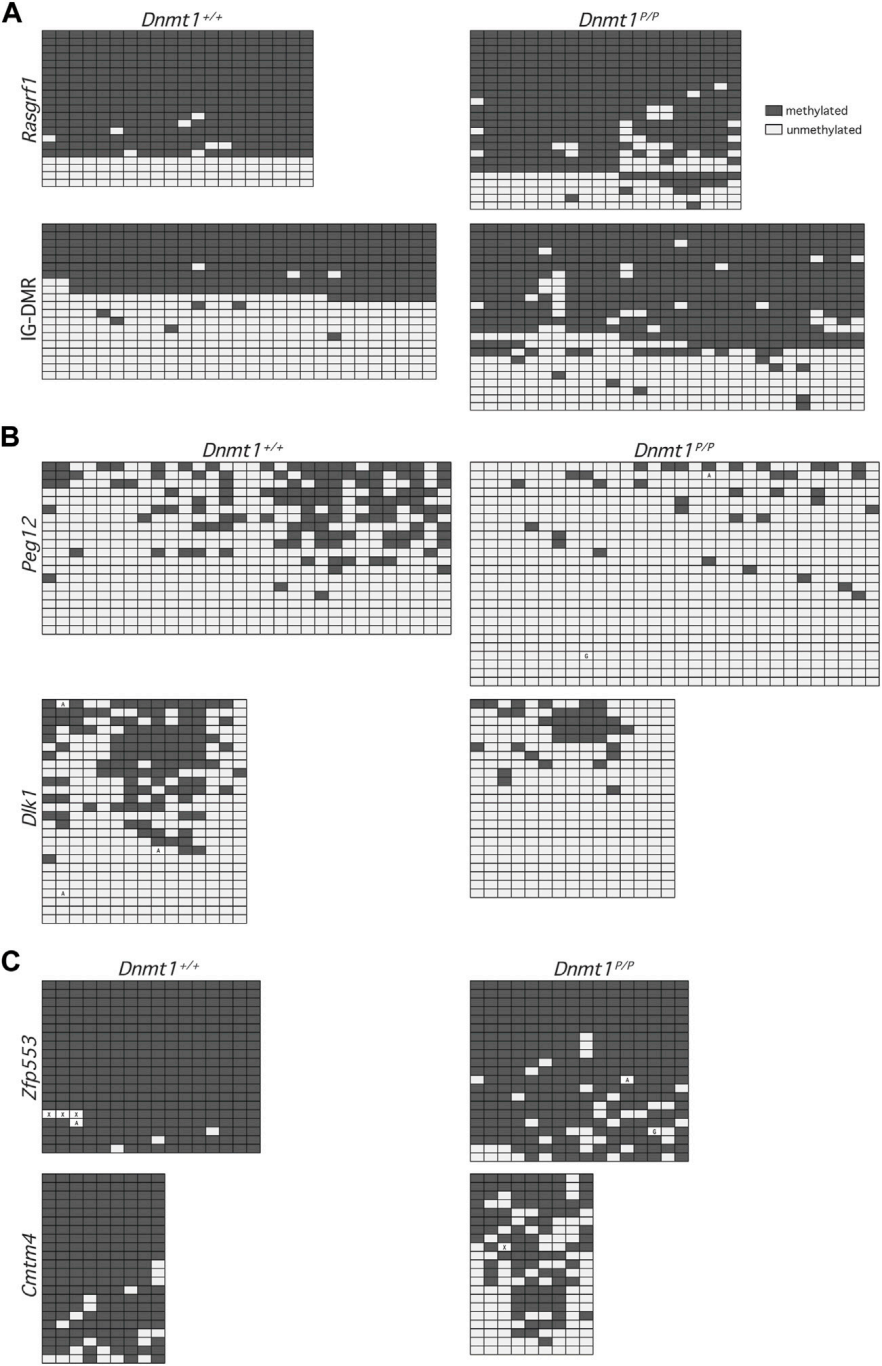
Methylation patterns across representative downsampled NGS sequences. Each row represents methylation data obtained at CpG dinucleotides within a single sequence: methylated (filled), unmethylated (open). Boxes containing an A or G represent PCR-induced error and indicate the nucleotide observed at that position; boxes containing an X represent undetermined sequence. Data were obtained from 12.5 dpc *Dnmt1* ^*+/+*^ (left) and *Dnmt1* ^*P/P*^ (right) embryos. **(A)** Primary DMRs *Rasgrf1* and IG-DMR. **(B)** Secondary DMRs *Peg12* and *Dlk1*. **(C)** Non-imprinted loci *Zfp553* and *Cmtm4*.

DNA methylation levels were reduced in *Dnmt1* ^*P/P*^ embryos at all loci examined and at all developmental stages analyzed relative to their wild-type siblings. At primary DMRs, the amount of methylation detected in *Dnmt1* ^*P/P*^ DNA was between 80% and 100% of the wild-type value at 87% of the sequences analyzed (Figure 2A; Supplementary Table S2). While all primary DMRs except the *Airn* ICR consistently exhibited reduced levels of DNA methylation in *Dnmt1* ^*P/P*^ mutant embryos, the difference in methylation between *Dnmt1* ^*+/+*^ and *Dnmt1* ^*P/P*^ embryos was generally less than 16%, suggesting either that the P allele form of Dnmt1 functions reasonably well at these sequences or that an alternative mechanism for maintaining methylation at these sequences exists. Furthermore, the amount of methylation observed at primary DMRs in wild-type and *Dnmt1* ^*P/P*^ embryos was consistent in biological replicates (Supplementary Figure S3A) and throughout the embryonic stages analyzed (Figure 2A; Supplementary Table S2). The observation that there is some loss of methylation at primary DMRs in *Dnmt1* ^*P/P*^ embryos suggests that methylation is imperfectly maintained at these sequences during early embryonic development. However, as additional loss of methylation was not observed in *Dnmt1* ^*P/P*^ mutant embryos as development progressed, whatever deficit the Dnmt1 P allele has in maintaining methylation occurs early and does not accumulate. The *H19* ICR displayed more dramatic differences in DNA methylation between the wild-type and *Dnmt1* ^*P/P*^ samples than the other primary DMRs analyzed, accounting for three of the four primary DMR samples where methylation maintenance was below 80% in *Dnmt1* ^*P/P*^ mutant embryos (Supplementary Table S2). This could be attributed to the fact that the *H19* ICR sample sizes were consistently very small and as a result the data obtained for this locus may not as accurately reflect the DNA methylation patterns present (Supplementary Table S3). Similarly, the observation that the amount of methylation at *Airn* is higher in *Dnmt1* ^*P/P*^ samples as compared to their wild-type siblings is likely an artifact associated with the small sample size. We consistently detected methylation levels around 70% for two of the primary DMRs analyzed, *Rasgrf1* and *Kcnq1ot1*, a higher value than would be expected based on their known parent of origin-specific methylation patterns (Figure 2A; Figure 3A). We believe this is likely due to biased amplification of the methylated allele at these loci, which appears to be occurring at the same frequency in the wild-type and *Dnmt1* ^*P/P*^ embryos (Figure 3A). Methylation levels at the remaining DMRs associated with imprinted loci were detected at expected frequencies in wild-type embryos.

To assess methylation levels at additional primary DMRs in wild-type vs. *Dnmt1* ^*P/P*^ embryos, we analyzed the 15.5 dpc RRBS data generated by Shaffer *et al.* (Shaffer et al., 2015). 17 of the 22 ICRs we analyzed were not represented in the tiles generated in their analysis, including all six of the primary DMRs targeted in our study. The RRBS data illustrated that methylation was well maintained at the primary DMRs associated with *Nespas/GnasXL*, *Inpp5f* and *Peg13*, with the percent methylation in *Dnmt1* ^*P/P*^ embryos being 89, 87% and 86% the level detected in wild-type embryos, respectively. Two ICRs, *Fkbp6* and *Cdh15*, showed more variation between wild-type and *Dnmt1* ^*P/P*^ embryos, with methylation differences of 24%.

In contrast to what was observed at primary DMRs, DNA methylation levels were dramatically reduced at secondary DMRs in *Dnmt1* ^*P/P*^ embryos (Figure 2B; Supplementary Figure S3B). At most secondary DMRs, the level of methylation in *Dnmt1* ^*P/P*^ embryos varied from 13% to 85% of the amount observed in their wild-type siblings. One notable exception to this finding was observed at *Igf2r*, which displayed a minimal reduction in methylation detected in wild type vs. *Dnmt1* ^*P/P*^ embryos. This difference could be attributed to the fact that while methylation is acquired at most secondary DMRs by 9.5 dpc, the secondary DMR associated with *Igf2r* acquires methylation during late gestation (Stöger et al., 1993; Bhogal et al., 2004; Gagne et al., 2014; Guntrum et al., 2017; Nechin et al., 2019). In support of this hypothesis, the average amount of methylation observed at *Igf2r* was approximately two-fold higher in 18.5 dpc embryos than in embryos collected at earlier developmental stages (Figure 2B; Supplementary Figure S3B). Excluding the *Igf2r* results, methylation levels in *Dnmt1* ^*P/P*^ embryos were below 80% the value observed in wild-type embryos at 84% of the sequences analyzed and below 50% in 49% of the sequences analyzed (Supplementary Table S2), considerably less than what was observed at the corresponding primary DMRs. Similar to what was observed at primary DMRs, methylation levels were relatively consistent across development suggesting that for the most part, methylation levels did not change in wild type nor in *Dnmt1* ^*P/P*^ embryos once it was acquired during early post-implantation development.

Shaffer *et al.* (Shaffer et al., 2015) illustrated a global loss of DNA methylation in *Dnmt1* ^*P/P*^ mice by analyzing methylation levels using methylation-sensitive Southern blots to examine methylation levels at IAP elements as well as LUMA assays to examine methylation levels across the genome. We took a targeted approach to examine DNA methylation levels at non-imprinted, single copy sequences. We analyzed methylation at two loci reported to have tissue-specific DNA methylation patterns, *Glut3* and *Hnf4a* (Yagi et al., 2008; Ganguly et al., 2014) as well as four ZFP57-bound loci displaying strain-specific methylation in embryonic stem cells: *Zfp553, Qrsl1, Cmtm4* and *Talpid3* (Strogantsev et al., 2015). All of the non-imprinted loci showed a reduction in the amount of DNA methylation present in *Dnmt1* ^*P/P*^ embryos as compared to their wild type siblings (Figure 2C), but the extent to which DNA methylation was lost varied between loci. Methylation was reasonably well maintained at *Zfp553* and *Talpid3*, but was dramatically reduced at *Glut3, Hnf4a, Cmtm4* and *Qrsl1*. Examination of these sequences using the CpG Island Finder (Gardiner-Garden and Frommer, 1987; EMBOSS, 2023), and the UCSC Genome Browser (UCSC Genome Browser) illustrated that CpG density varies at the loci examined in our study. All six primary DMRs and all nine secondary DMRs contain CpG islands or are CpG-rich. In contrast, the regions of *Zfp553*, *Glut3* and *Hnf4a* analyzed are CpG-rich, but the methylated regions of *Cmtm4*, *Qrsl1* and *Talpid3* examined in this study are CpG-poor (Supplementary Figure S4A). Therefore, CpG density does not correlate with the ability of the *Dnmt1* P allele to maintain methylation.

We further investigated these loci to determine whether *Zfp553* and *Talpid3*, non-imprinted sequences that retain methylation in *Dnmt1* ^*P/P*^ mutant embryos, share any features with primary DMRs that might make them resistant to methylation loss. While *Zfp553* and *Talpid3* display methylation in embryonic stem cells that is presumably gametic in origin (Strogantsev et al., 2015), so do *Qrsl1* and *Cmtm4*, which show dramatic loss of methylation in *Dnmt1* ^*P/P*^ mutant embryos (Figure 2C). Furthermore, Shaffer and others (Shaffer et al., 2015) illustrated that methylation is lost at gametically methylated IAP elements in *Dnmt1* ^*P/P*^ mutant embryos. Together, these data suggest that gametic inheritance of methylation does not predict a sequence’s ability to retain methylation in the presence of the P allele form of Dnmt1. We additionally assessed each locus for other chromatin features, including euchromatin vs. heterochromatin status, enhancer vs. promoter vs. transcription unit status, the presence of the transcriptionally permissive histone modifications H3K4me1, H3K4me3 and H3K27ac, and the presence of histone modifications associated with transcriptional repression and/or DNA methylation, H3K9me3, H3K36me3 and H3K27me3, in 12.5 dpc mouse midbrain using the UCSC Genome Browser (Supplementary Figure S4B). While both *Zfp553* and *Talpid3* are enriched for H3K36me3, the only primary DMR containing this modification is *Airn.* Based on these analyses, there was no apparent association between a particular chromatin signature and the ability of the corresponding sequence to retain methylation in *Dnmt1* ^*P/P*^ mutant embryos. Of note, many of the DMRs associated with imprinted loci displayed both permissive and repressive modifications, consistent with the fact that the parental alleles have opposing epigenetic states.

### 3.4 DNA methylation loss is randomly distributed across individual sequences in *Dnmt1* ^*P/P*^ embryos

The lower levels of DNA methylation observed in *Dnmt1* ^*P/P*^ embryos could be due to loss of methylation across a subset of DNA strands, loss of methylation at specific sequences within the DNA, or non-specific loss of methylation across all DNA strands. To distinguish between these possibilities, we extracted a subset of the NGS sequences obtained and analyzed the DNA methylation profiles of individual sequences. Analysis of the extracted primary DMR sequences for *Rasgrf1* and the *Dlk1-Dio3* IG-DMR showed that the methylation profile amongst alleles obtained from *Dnmt1* ^*P/P*^ embryos was not significantly different than it was in their wild-type siblings (*p* values = 0.1802, 0.4839), and further illustrated that methylation was generally well maintained across the sequences analyzed (Figure 3A; additional data not shown). In contrast, the methylation patterns observed at the secondary DMRs associated with *Dlk1* and *Peg12* in wild type vs. *Dnmt1* ^*P/P*^ embryos showed significant loss of methylation across each locus (*p* values = 0.0041, 0.0088), with inconsistent methylation remaining (Figure 3B; additional data not shown). Similar trends were observed at non-imprinted loci that showed either modest or dramatic differences in methylation between wild type and *Dnmt1* ^*P/P*^ embryos (Figure 3C). Methylation was better maintained at *Zfp553* in *Dnmt1* ^*P/P*^ embryos and was distributed evenly across the sequences analyzed, although the loss of methylation between wild type and *Dnmt1* ^*P/P*^ embryos was significant (*p*-value = 0.0005). *Cmtm4* displayed a dramatic and dispersed loss of methylation in *Dnmt1* ^*P/P*^ embryos (*p*-value = <0.0001). Overall, these data suggest that methylation is poorly maintained across loci in *Dnmt1* ^*P/P*^ embryos rather than lost entirely from specific sequences.

## 4 Discussion

DNA methyltransferases carry out both *de novo* and maintenance methylation, with Dnmt1 primarily functioning as the maintenance methyltransferase and *de novo* methylation resulting from the activity of Dnmt3a and Dnmt3b (Li and Zhang, 2014; Hervouet et al., 2018). The fidelity of Dnmt1 in maintaining methylation patterns by methylating newly synthesized daughter strands has been estimated to be 95%–96% (Ushijima et al., 2003; Laird et al., 2004; Vilkaitis et al., 2005), yet high levels of hemimethylation have been observed at some genomic loci, including secondary DMRs associated with imprinted genes, suggesting inconsistent maintenance of methylation at these sequences (Guntrum et al., 2017; Nechin et al., 2019). We previously found that 30%–50% of the CpG dyads in secondary DMRs are hemimethylated, suggesting that DNA methylation is passively and/or actively lost at these sequences, possibly due to 5-hydroxymethylcytosine enrichment at these sequences (Guntrum et al., 2017; Nechin et al., 2019) (TDavis lab, data not shown). Despite the fact that hemimethylation should result in reduced methylation levels following subsequent rounds of DNA replication, methylation levels remain constant at secondary DMRs throughout development, leading us to propose that methylation at these loci may be lost due to reduced Dnmt1 fidelity and restored by *de novo* methylation via Dnmt3a/3b. Indeed, despite the established roles of Dnmt1 and the Dnmt3 family proteins, evidence suggests that Dnmt3 enzymes may function cooperatively with Dnmt1 to maintain methylation at repetitive and CpG-rich sequences (Liang et al., 2002; Chen et al., 2003; Jones and Liang, 2009; Liu et al., 2022). While Dnmt1 appears to be sufficient for maintaining DNA methylation in ES cells at many primary DMRs associated with imprinted genes, Dnmt3a and 3b contribute to maintenance methylation at several ICRs including *H19* and IG-DMR and Dnmt3b has been shown to be necessary for maintaining methylation at *Rasgrf1* (Hirasawa et al., 2008; Liu et al., 2022). Liu and others (Liu et al., 2022) further suggested that Dnmt3a and 3b may be more important than Dnmt1 for maintaining methylation at approximately half of the secondary DMRs analyzed in their study.

The suggestion that Dnmt3a/3b may function cooperatively with Dnmt1 in maintaining methylation raises a question as to what directs Dnmt3a and/or Dnmt3b to methylate secondary DMRs and other loci throughout development. We hypothesized that primary DMRs signal the *de novo* acquisition of methylation at secondary DMRs in the same imprinting cluster, and that this activity occurs both during the initial acquisition of methylation at secondary DMRs during post-implantation development and throughout the remainder of development. Several lines of evidence support this hypothesis, best illustrated at the *Dlk1*-*Dio3* imprinting cluster. Analysis of methylation and gene expression patterns in patients with IG-DMR and *MEG3*-DMR microdeletions illustrate the hierarchical way in which DNA methylation is established across this imprinting cluster (Kagami et al., 2010; Beygo et al., 2015). Furthermore, deletion of the tandem repeat in the paternally-inherited IG-DMR, its replacement with CpG-free sequences and targeted demethylation of the repeat via epigenetic editing in mice all resulted in loss of methylation at the IG- and *Gtl2*-DMRs and concomitant loss of imprinting at both maternally- and paternally-expressed imprinted genes (Saito et al., 2018; Hara et al., 2019; Aronson et al., 2021; Kojima et al., 2022). These experiments demonstrated that the methylation status of the tandem repeat within the ICR is necessary both to establish and maintain parental epitypes and expression profiles across this cluster.

If the methylation status at the primary DMR is the primary driver of the methylation status at the corresponding secondary DMRs within the same imprinting cluster, then methylation should be maintained equally well at primary and secondary DMRs in *Dnmt1* ^*P/P*^ mutant mice, but our results did not support this hypothesis. Despite the fact that embryos homozygous for the *Dnmt1* P allele maintain methylation relatively well at primary DMRs associated with imprinted genes (data herein and (Shaffer et al., 2015)), we found that methylation was dramatically reduced at secondary DMRs in *Dnmt1* ^*P/P*^ embryos as compared to their wild-type siblings. Therefore, while we cannot exclude the possibility that the Dnmt3 proteins work cooperatively with Dnmt1 in maintaining methylation at secondary DMRs, their action cannot compensate for the mutant Dnmt1 protein. This could be because while wild-type Dnmt1 interacts with Dnmt3a/b (Kim et al., 2002; Qin et al., 2011), mutant Dnmt1 is unable to do so either because of its disrupted structure and/or its low concentration (Shaffer et al., 2015). Failure of such an interaction could impact the maintenance of methylation that requires the coordinated activity of both Dnmt1 and Dnmt3 proteins. To further explore whether the Dnmt3 proteins play a role in maintaining methylation at imprinted loci, it will be important to assess whether either or both of these enzymes localize to primary and/or secondary DMRs in wild-type and *Dnmt1* ^*P/P*^ mutant embryos at developmental stages after methylation is initially established.

The *Dnmt1* P allele has dramatically different effects at different loci within the mouse genome. This mutation is located within an N-terminal intrinsically disordered domain that interacts with at least 8 different proteins that may play roles in regulating Dnmt1 activity both broadly and at specific sequences (Liu et al., 2017). In support of this hypothesis, different mutations within the IDD impact DNA methylation in different ways, with some mutations affecting methylation at ICRs but having no effect on non-ICR methylation while other mutations have the opposite effect (Borowczyk et al., 2009; Shaffer et al., 2015). The ability of different, expressed, IDD-deleted forms of Dnmt1 protein to selectively maintain DNA methylation at imprinted vs. non-imprinted sequences supports the idea that IDD-mediated protein-protein interactions provide specificity to Dnmt1 activity (Borowczyk et al., 2009). Shaffer and others (Shaffer et al., 2015) suggested that the region altered in the P allele, which is specific to *Mus* and *Rattus*, might be important for methylation of species-specific sequences. The P allele mutation results in a local increase in the intrinsic disorder score which likely impacts the way in which Dnmt1 interacts with other proteins (Liu et al., 2017) and may therefore affect the ability of Dnmt1 to interact efficiently with proteins that generally guide it to hemimethylated DNA, such as UHRF1 and MeCP2 (Kimura and Shiota, 2003; Bostick et al., 2007; Sharif et al., 2007; Zhang et al., 2011). While alteration of these sequences may disrupt protein-protein interactions and Dnmt1 activity, Shaffer *et al.* also illustrated that Dnmt1 protein levels are dramatically reduced in *Dnmt1* ^*P/P*^ mutant mid-to late gestation embryos and suggested that failure of the P allele mutant Dnmt1 protein to interact with other proteins may lead to its degradation, compromising its ability to methylate newly replicated sequences (Shaffer et al., 2015). Since global methylation is significantly decreased in *Dnmt1* ^*P/P*^ mid-to late gestation embryos, but ICR methylation is maintained ((Shaffer et al., 2015) and data herein), it is possible that Dnmt1 activity at ICRs is less dependent on the region disrupted by the P allele because the mechanism by which Dnmt1 maintains methylation at these sequences is different than the mechanism it uses to more generally maintain methylation across the mouse genome. Alternatively, Dnmt1 may have a higher affinity for ICR sequences, resulting in its activity primarily being directed to those genomic regions even when protein levels are low. It is also possible that methylation at primary DMRs is maintained in an alternate way in *Dnmt1* ^*P/P*^ individuals, perhaps through the action of Dnmt3a/3b. In support of this hypothesis, Thakur and others (Thakur et al., 2016) demonstrated the ability of a Dnmt3a isoform to restore methylation at primary DMRs in *Dnmt3a/3b* knock-out ES cells.

Given the dispersed pattern of methylation at imprinted and non-imprinted loci in *Dnmt1* ^*P/P*^ embryos, we suggest that methylation fidelity is reduced in the presence of this mutation because the mutant Dnmt1 fails to faithfully recognize hemimethylated sequences and methylate the newly synthesized complement, thereby leading to an overall loss of methylation. Preliminary data from our lab illustrates an increase in hemimethylation in sequences derived from *Dnmt1* ^*P/P*^ embryos: we found significantly more hemimethylation at the IG-DMR in *Dnmt1* ^*P/P*^ 12.5 dpc embryos as compared to their wild-type siblings (18.34% vs. 12.89%, *p* = 0.0407; data not shown). Additional analyses will be necessary to further test this hypothesis.

Despite the dramatic loss of global methylation in *Dnmt1* ^*P/P*^ mice, embryonic development appears to progress relatively normally although *Dnmt1* ^*P/P*^ individuals are unable to survive after birth, presumably as a consequence of altered gene expression patterns. While DNA methylation at promoters correlates with gene silencing (Li and Zhang, 2014), the precise amount of promoter DNA methylation required to achieve silencing at individual loci has not been studied in detail and is likely not generalizable. It is known that loss of Dnmt1 activity has a dramatic impact on imprinted gene expression (Li et al., 1993; Caspary et al., 1998; Nakagaki et al., 2014), but in these mutants methylation is dramatically reduced at both primary and secondary DMRs, complicating the ability to determine how loss of methylation specifically at secondary DMRs, without altering their primary sequence, impacts imprinted gene expression. The differential effects of the P allele on methylation levels at primary vs. secondary DMRs associated with imprinted genes provides an opportunity for assessing the relative importance of methylation at primary vs. secondary DMRs in regulating the expression of individual imprinted genes, and these experiments are currently underway.

## Data Availability

The datasets presented in this study can be found in online repositories. The names of the repository/repositories and accession number(s) can be found below: NCBI SRA under the following accession numbers SAMN34130389, SAMN34130390, SAMN34130391, SAMN34130392, SAMN34130393, SAMN34130394, SAMN34130395, SAMN34130396, SAMN34130397, SAMN34130398.
