# Supplementary material for: Methylation is maintained specifically at imprinting control regions but not other DMRs associated with imprinted genes in mice bearing a mutation in the Dnmt1 intrinsically disordered domain
Source: Front Cell Dev Biol. 2023 Aug 4;11:1192789. doi: 10.3389/fcell.2023.1192789 (PMC10436486; doi:10.3389/fcell.2023.1192789)
Supplement: Supplementary file 2 [file Table1.docx]

**Supplementary Table 1**: PCR conditions

| DMRs associated with imprinted loci | | | | | |
| --- | --- | --- | --- | --- | --- |
| Locus;  1° or 2° DMR | **Round** | **Forward Primer and Sequence** | **Reverse Primer and Sequence** | **Annealing temperature** | **Size (bp)** |
| *Dlk1*-DMR  2° | 1 | DlkBisF1:  CTATTTCTACTACTCTATCCTAACC | DlkBisR1:  GTGTTTAGTATTATTAGGTTGGTGA | 50°C |  |
|  | 2 | DlkBisF2:  CCCTAA CTTCTCAAACAACTTCTA | DlkBisR2:  GGATGGTGAAGTAGATGGTTTGT | 52°C | 458 |
| *Gtl2*-DMR  2° | 1 | Gtl2BisF1: GTAGAGATTTTGATTAAGAAAAGTAG | Gtl2BI4R1:  CAACACTCAAATCACCCCCC | 52°C |  |
|  | 2 | Gtl2BisF2:  GGGTTTAAAAATTTGGTTTTGGTG | Gtl2BI4R2:  GCCCCCCACATCTATTCTACC | 52°C | 412 |
| *H19*ppDMR  2° | 1 | H19pp-oxBS-F3: GAAGGATTATTAGGAGTTAGAAATGAG | H19pp-oxBS-R2: ACTAACCCCCAACCTTTATCCTAATCT | 55°C |  |
|  | 2 | H19pp-NEW-F: GTGAGAATTATTGTTGAGTGGTTATG | H19pp-oxBS-R2: ACTAACCCCCAACCTTTATCCTAATCT | 55°C | 463 |
| *H19* ICR  1° | 1 | BMsp2t1:  GAGTATTTAGGAGGTATAAGAATT | BHha1t3:  ATCAAAAACTAACATAAACCCCT | 55°C |  |
|  | 2 | BMsp2t2:  GTAAGGAGATTATGTTTTATTTTTG | BHha1t4:  CCTCATAAAACCCATAACTAT | 55°C | 422 |
| IG-DMR  1° | 1 | IGoxBS-F3: GAGATGTGTTGTGGATTTAGGTTGTAG | IGoxBS-R2: CTATACTCAAAACATTCTCCATTAACAA | 58°C |  |
|  | 2 | IGoxBS-F4: AGGTTGATTATGTATAAGTGTTGTGG | IGoxBS-R1: CAACCCTTCCCTCACTCCAAAAATT | 52°C | 343 |
| *Airn*  1° | 1 | AirnBS-F3:  GAGAGGTTAAGGGTGAAAAGTTG | AirnBS-R3: CACTTTTAAATTCATCTCTCCTATAAC | 55°C |  |
|  | 2 | AirnBS-F4:  GTGAAAAGTTGTATAAGGAGGGG | AirnBS-R4: CTCTCCTATAACATAACACTTTTAAAC | 55°C | 434 |
| *Igf2r-*DMR  2° | 1 | Igf2r-DMR1-BS-F2: GAGGAGAGAGAATTGGAGTTAGG | Igf2r-DMR1-BS-R1: AAACTAACCTCTTAACCCTACATC | 52°C |  |
|  | 2 | Igf2r-DMR1-BS-F2: GAGGAGAGAGAATTGGAGTTAGG | Igf2r-DMR1-BS-R2: CTACATCCACTTACAACACTAAAC | 52°C | 425 |
| *Kcnq1ot1*  1° | 1 | Lit1-BFo: GTGTGATTTTATTTGGAGAGTTTTTTTG | Lit1-BRo-new: CCACTCACTACCTTAATACTAACCAC | 50°C |  |
|  | 2 | Lit1-BFi: GTTTGGAGAGTTTTTTTGTTTAGTTTGG | Lit1-BRi-new: ATCCTAAACCACTCACCTTAAAAC | 50°C | 434 |
| *Magel2-*DMR  2° | 1 | Magel2-BS-F1: TAAGTTAGGTAGTAGTATTTGGTTG | Magel2-BS-R1: CTAAACCATTAAAACCCCTAAACC | 50°C |  |
|  | 2 | Magel2-BS-F2: GGTAGTGTTTGTTGAGAGTTGTTG | Magel2-BS-R2: AACTAAACCAACTAAACCACTAAAAC | 55°C | 447 |
| *Mkrn3­*-DMR  2° | 1 | Mkrn3BS-F1: ATGTTTGTATTGTTAATTTTAGTTTTAG | Mkrn3BS-R: CCTCAATTTAAAACTACTAAAACTTC | 50°C |  |
|  | 2 | Mkrn3BS-F2: GTTAATTAGAGGTAAGGAGTTTGAG | Mkrn3BS-R: CCTCAATTTAAAACTACTAAAACTTC | 50°C | 414 |
| *Ndn*-DMR  2° | 1 | NdnB-F1:  TTATTTAGTTTTGTGTTATATAGGAG | NdnB-R1:  CCTTCTACACCAACTAAACAAAA | 50°C |  |
|  | 2 | NdnB-F3:  AGATTTTAGTGGTTGGGTTTTGT | NdnB-R3:  CCTATTAAACTACCATAAAACCTT | 52°C | 394 |
| *Cdkn1c*-DMR  2° | 1 | p57U3BFO:  GTATTGTTAGGATTAGGATTTAGTTG | p57U3BR:  GTAACATAATATAATATTTTCAATTTCAAC | 45°C |  |
|  | 2 | p57U3BFI:  GTTTTAATTAGTTGGTGTAGTTTTAGG | p57U3BRi:  CCCACAAAAACCCTACCCC | 45°C | 375 |
| *Peg12*-DMR  2° | 1 | Peg12Bis-F:  ACTCCTACAACAACTCCTTCTT | Peg12BM-F1:  TTGTTATAGGTTGGTGTTGTGG | 52°C |  |
|  | 2 | Peg12Bis-F:  ACTCCTACAACAACTCCTTCTT | Peg12BM-F2: GGTTATTAGTTGTTAGGGATAAG | 52°C | 245 |
| *Rasgrf1*  1° | 1 | RasBF1:  GAGAGTATGTAAAGTTAGAGTTGG | RasBR: ATAATACAACAACAACAATAACAATC | 52°C |  |
|  | 2 | RasBF2: GTTAAAGATAGTTTAGATATGGAATTT | RasBR: ATAATACAACAACAACAATAACAATC | 55°C | 252 |
| *Snrpn*  1° | 1 | Snrpn-B: AATTTGTGTGATGTTTGTAATTATTTGG | Snrpn-OxBs-R4: CTCCAAAAAATTACTCACCAATTCTC | 55°C |  |
|  | 2 | Snrpn-OxBs-F4: ATTATATTTATTATTTTAGATTGATAGTG | Snrpn-C: ATAAAATACACTTTCACTACTAAAATCC | 52°C | 352 |
| Promoters associated with non-imprinted loci | | | | | |
| Locus | **Round** | **Forward Primer and Sequence** | **Reverse Primer and Sequence** | **Annealing temperature** | **Size (bp)** |
| *Glut3* | 1 | Glut3-oxBS-F1:  TTGTAGGAGGTAGGTATATTTTAGG | Glut3-HPbisR1:  CACCAAATAACTTAAATTCTCTTTCTAC | 55°C |  |
|  | 2 | Glut3-oxBS-F2:  GGTGAGTTTTAGAGTGAAGTAGTTG | Glut3-HPbisR2:  CTCTTTCTACTAACAAACACTAACCTTT | 55°C | 437 |
| *Hnf4a* | 1 | Hnf4a-BS-F2: TATTGTGGTTAGAGGTATTTTGGG | Hnf4a-oxBS-R1:  AATCTATCATCCACATTTACAAACAC | 52°C |  |
|  | 2 | Hnf4a-BS-F3:  GTGGTTGTAGAAATTAATTTTGTTTA | Hnf4a-oxBS-R2:  CCTAAAAACAAAACTACAAACCATTC | 52°C | 347 |
| *Zfp553* | 1 | Zfp-oxBS-F1: GAGAAGTTTTATAAGTGTGGTATTTG | Zfp-HP-R1: CTATCAACAAAACCTTTACCACACs | 50°C |  |
|  | 2 | Zfp-oxBS-F2: GTAAGGGTTTTGGGGATAGTTTAG | Zfp-HP-R2: CAAATAAAACTTCTCACCTATATATAC | 50°C | 319 |
| *Cmtm4* | 1 | Cmtm4-oxBS-F1:  GGAGATTGTAGGTGTTGTATATTG | Cmtm4-HP-R1:  CCTAATTCTTAAATATACTATCAAACC | 52°C |  |
|  | 2 | Cmtm4-oxBS -F2:  GTGTTGTATATTGTTTGGATTGGTG | Cmtm4-HP -R2:  CCCACCCCTACTTCCCAACAC | 55°C | 284 |
| *Qrsl1* | 1 | Qrsl-oxBS-F1: AAGGGAAATTATGGTAATTGATTTGG | Qrsl-oxBS-R1: TATATTTTTAAACACAAACAACTCACC | 55°C |  |
|  | 2 | Qrsl-oxBS-F2: GTAATTGATTTGGATAATTAGTTGGG | Qrsl-oxBS-R2: CTAAATTCCTATACCACTAAACTTTAC | 50°C | 416 |
| *Talpid3* | 1 | Talpid3-F1:  GTTGTTTAGTATTTATTAGTGAGTAG | Talpid3-R3:  aatactcaaacaaaaccactatctCc | 50°C |  |
|  | 2 | Talpid3-F2: TGTTTATAGATGTATTTTTATTGAGAG | Talpid3-R4: AAAAACTACAAACCACTACAACAACC | 52°C | 330 |
