# Supplementary material for: Methylation is maintained specifically at imprinting control regions but not other DMRs associated with imprinted genes in mice bearing a mutation in the Dnmt1 intrinsically disordered domain
Source: Front Cell Dev Biol. 2023 Aug 4;11:1192789. doi: 10.3389/fcell.2023.1192789 (PMC10436486; doi:10.3389/fcell.2023.1192789)
Supplement: Supplementary file 3 [file Image4.pdf]

A

CpG density at primary DMRs, secondary DMRs and non-imprinted loci

	CpG island	CpG-rich	CpG-poor
**Primary DMRs**	IG-DMR, *Airn*, *Kcnq1ot1*	*H19* ICR, *Rasgrf1*, *Snrpn*	
**Secondary DMRs**	*Dlk1*, *Peg12*, *Ndn*, *Magel2*, *Mkrn3*, *Igf2r*, *Cdkn1c*	*H19*pp DMR, *Gtl2*	
**Non-imprinted loci**		*Zfp553*, *Hnf4a, Glut3*	*Qrsl1*, *Cmtm4*, *Talpid3*

B

Chromatin signatures at primary DMRs, secondary DMRs and non-imprinted loci

	Chromatin status				H3K4me1	H3K4me3	H3K27ac
	heterochromatin	enhancer	promoter	transcription unit			
**Primary DMRs**	IG-DMR	*H19* ICR	*Snrpn*, *Airn*, *Kcnq1ot1*		*H19* ICR, IG-DMR	*H19* ICR, IG-DMR, *Rasgrf1*, *Snrpn*, *Airn*, *Kcnq1ot1*	*Snrpn*, *Kcnq1ot1*
**Secondary DMRs**		*Dlk1*	*H19*pp DMR, *Gtl2, Peg12*, *Ndn*, *Magel2*, *Mkrn3*, *Igf2r*, *Cdkn1c*		*Dlk1, Igf2r*	*H19*pp DMR, *Gtl2, Dlk1, Peg12*, *Ndn*, *Magel2*, *Mkrn3*, *Igf2r*, *Cdkn1c*	*Gtl2, Peg12*, *Ndn*, *Magel2*, *Mkrn3*, *Igf2r*, *Cdkn1c*
**Non-imprinted loci**	*Hnf4a*		*Glut3*	*Qrsl1*, *Cmtm4*, *Talpid3, Zfp553*		*Glut3*	

Supplementary Figure S4. Chromatin signatures do not correlate with retention of methylation in *Dnmt1*$^{P/P}$ mutant embryos. **(A)** CpG density at primary DMRs, secondary DMRs and non-imprinted loci. **(B)** Chromatin signatures at primary DMRs, secondary DMRs and non-imprinted loci.
