# Supplementary material for: Methylation is maintained specifically at imprinting control regions but not other DMRs associated with imprinted genes in mice bearing a mutation in the Dnmt1 intrinsically disordered domain
Source: Front Cell Dev Biol. 2023 Aug 4;11:1192789. doi: 10.3389/fcell.2023.1192789 (PMC10436486; doi:10.3389/fcell.2023.1192789)
Supplement: Supplementary file 4 [file Image2.pdf]

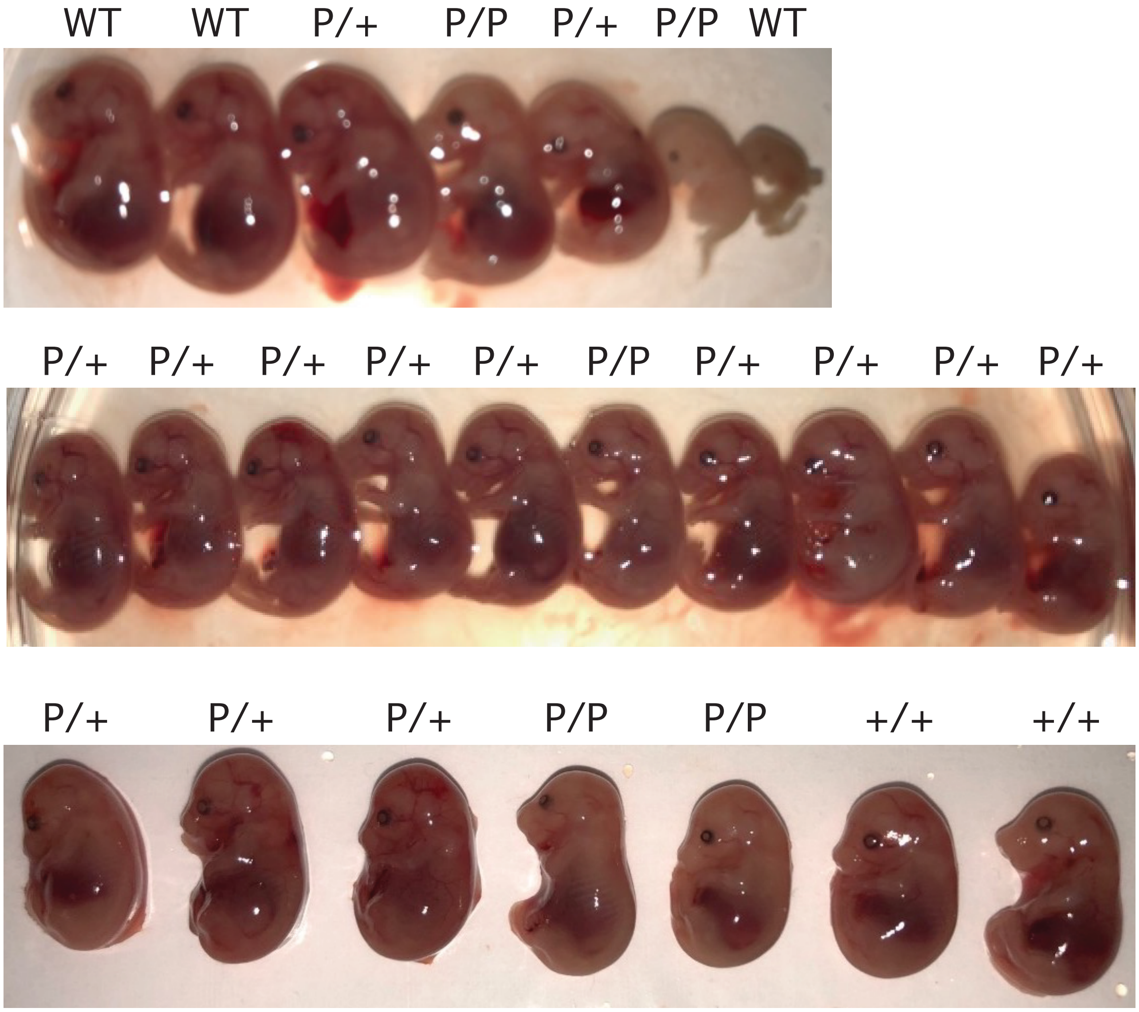

Supplementary Figure S2. Comparison of 15.5 dpc Dnmt1+/+ (WT), Dnmt1P/+ (P/+) and Dnmt1P/P (P/P) embryos. Three 15.5 dpc litters collected from separate Dnmt1P/+ x Dnmt1P/+ natural matings illustrate that there were no consistent morphological differences between WT, P/+ and P/P embryos.
