# Supplementary material for: Methylation is maintained specifically at imprinting control regions but not other DMRs associated with imprinted genes in mice bearing a mutation in the Dnmt1 intrinsically disordered domain
Source: Front Cell Dev Biol. 2023 Aug 4;11:1192789. doi: 10.3389/fcell.2023.1192789 (PMC10436486; doi:10.3389/fcell.2023.1192789)
Supplement: Supplementary file 5 [file Image3.pdf]

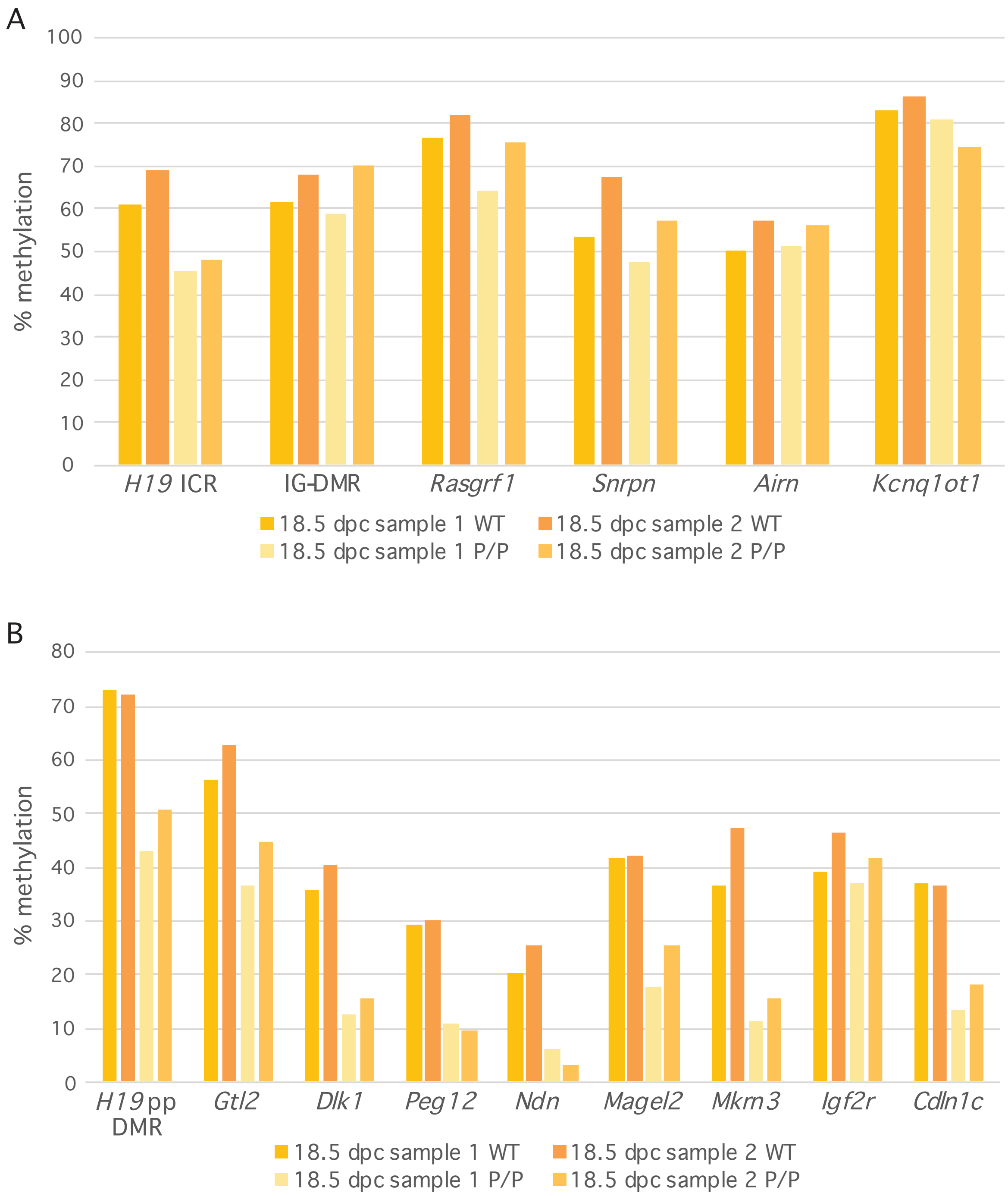

Supplementary Figure S3. Methylation levels are similar in 18.5 dpc biological replicates. Percent methylation derived at each locus from NGS data; data from two 18.5 dpc Dnmt1+/+ (WT) and two Dnmt1P/P (P/P) embryos. (**A**) Primary DMRs. (**B**) Secondary DMRs.
