# Supplementary material for: Methylation is maintained specifically at imprinting control regions but not other DMRs associated with imprinted genes in mice bearing a mutation in the Dnmt1 intrinsically disordered domain
Source: Front Cell Dev Biol. 2023 Aug 4;11:1192789. doi: 10.3389/fcell.2023.1192789 (PMC10436486; doi:10.3389/fcell.2023.1192789)
Supplement: Supplementary file 6 [file Table3.docx]

**Supplementary Table 3**. Sample size of data obtained from NGS analysis.

| Gene/locus | Maximum number of strands extracted | | | | | | | | | |
| --- | --- | --- | --- | --- | --- | --- | --- | --- | --- | --- |
| stage | **9.5 dpc** | | **12.5 dpc** | | **15.5 dpc** | | **18.5 dpc sample 1** | | **18.5 dpc sample 2** | |
| genotype | **WT** | **P/P** | **WT** | **P/P** | **WT** | **P/P** | **WT** | **P/P** | **WT** | **P/P** |
| *H19* ICR | 1903 | 1918 | 31 | 30 | 9 | 4 | 262 | 1138 | 65 | 160 |
| IG-DMR | 12495 | 5548 | 6282 | 7513 | 939 | 901 | 3217 | 2554 | 2816 | 2338 |
| *Rasgrf1* | 53607 | 40400 | 40848 | 22960 | 16608 | 14060 | 2624 | 5272 | 20070 | 50502 |
| *Snrpn* | 1029 | 308 | 399 | 247 | 667 | 598 | 6897 | 986 | 78 | 162 |
| *Airn* | 5579 | 1685 | 2212 | 1118 | 40 | 52 | 107 | 1986 | 1051 | 606 |
| *Lit1* | 1943 | 1991 | 722 | 773 | 958 | 569 | 2176 | 1244 | 127 | 100 |
| *H19*pp-DMR | 496 | 545 | 1128 | 2518 | 49 | 52 | 222 | 115 | 982 | 280 |
| *Gtl2* | 1693 | 630 | 511 | 118 | 51 | 35 | 139 | 70 | 42 | 19 |
| *Dlk1* | 15567 | 6166 | 1470 | 1574 | 1264 | 964 | 2130 | 1027 | 15953 | 8055 |
| *Peg12* | 21816 | 10492 | 1771 | 1448 | 5499 | 3934 | 779 | 395 | 3594 | 4990 |
| *Ndn* | 7118 | 6137 | 1473 | 1080 | 470 | 570 | 2482 | 922 | 120 | 67 |
| *Magel2* | 3087 | 1670 | 2164 | 1091 | 3037 | 1606 | 3456 | 761 | 1649 | 2237 |
| *Mkrn3* | 7550 | 7007 | 846 | 782 | 1168 | 1293 | 3838 | 2785 | 1588 | 1441 |
| *Igf2r* | 7770 | 7750 | 1447 | 2269 | 3360 | 3663 | 799 | 392 | 4242 | 2359 |
| *Cdkn1c* | 174 | 117 | 13 | 7 | 7 | 6 | 87 | 255 | 49 | 71 |
| *Glut3* | ND | ND | 1318 | 3100 | ND | ND | ND | ND | 3739 | 1500 |
| *Hnf4a* | ND | ND | 1593 | 1857 | ND | ND | ND | ND | ND | ND |
| *Zfp553* | ND | ND | 9693 | 8280 | ND | ND | ND | ND | 58806 | 63277 |
| *Qrsl1* | ND | ND | 2147 | 1911 | ND | ND | ND | ND | 619 | 568 |
| *Cmtm4* | ND | ND | 8322 | 2952 | ND | ND | ND | ND | 11406 | 23986 |
| *Talpid3* | ND | ND | 2927 | 2079 | ND | ND | ND | ND | 2695 | 1821 |

Data presented represent the maximum number of strands extracted from each NGS dataset based on the largest number of CpGs analyzed at each locus. ND = not determined.

| Gene/locus | Total number of CpG sites extracted | | | | | | | | | |
| --- | --- | --- | --- | --- | --- | --- | --- | --- | --- | --- |
| stage | **9.5 dpc** | | **12.5 dpc** | | **15.5 dpc** | | **18.5 dpc sample 1** | | **18.5 dpc sample 2** | |
| genotype | **WT** | **P/P** | **WT** | **P/P** | **WT** | **P/P** | **WT** | **P/P** | **WT** | **P/P** |
| *H19* ICR | 28845 | 29602 | 367 | 366 | 76 | 55 | 4003 | 18032 | 821 | 1580 |
| IG-DMR | 357459 | 154489 | 178990 | 215318 | 25818 | 24918 | 89309 | 65683 | 80915 | 66880 |
| *Rasgrf1* | 1031687 | 790293 | 803342 | 447914 | 319758 | 271366 | 49750 | 97722 | 391989 | 988309 |
| *Snrpn* | 16158 | 4606 | 6291 | 3888 | 10274 | 9193 | 109240 | 14107 | 1030 | 2516 |
| *Airn* | 160831 | 47128 | 63435 | 31907 | 1059 | 1265 | 2925 | 55800 | 30059 | 16581 |
| *Lit1* | 66355 | 68337 | 23911 | 26263 | 33837 | 19922 | 76794 | 43305 | 3902 | 3151 |
| *H19*pp-DMR | 4626 | 4877 | 10925 | 24539 | 395 | 433 | 2180 | 1077 | 9321 | 2568 |
| *Gtl2* | 28298 | 9640 | 8889 | 2034 | 835 | 460 | 2073 | 530 | 537 | 201 |
| *Dlk1* | 450126 | 184021 | 43790 | 46577 | 36404 | 28588 | 63587 | 30647 | 475802 | 239792 |
| *Peg12* | 325589 | 154051 | 26423 | 21686 | 82321 | 58845 | 11470 | 5486 | 52592 | 74680 |
| *Ndn* | 194612 | 169587 | 38326 | 27909 | 12813 | 15409 | 66555 | 25226 | 2516 | 1331 |
| *Magel2* | 63783 | 33889 | 43782 | 22627 | 63099 | 33157 | 71265 | 15316 | 34016 | 45511 |
| *Mkrn3* | 194855 | 181523 | 21716 | 20124 | 30162 | 33506 | 99454 | 72213 | 40618 | 36580 |
| *Igf2r* | 298590 | 299692 | 55938 | 87850 | 130334 | 141845 | 29495 | 14460 | 163434 | 91146 |
| *Cdkn1c* | 5720 | 2268 | 466 | 125 | 142 | 144 | 1595 | 4020 | 1134 | 1612 |
| *Glut3* | ND | ND | 19476 | 46050 | ND | ND | ND | ND | 54981 | 21870 |
| *Hnf4a* | ND | ND | 18790 | 22092 | ND | ND | ND | ND | ND | ND |
| *Zfp553* | ND | ND | 154374 | 131919 | ND | ND | ND | ND | 939063 | 1009996 |
| *Qrsl1* | ND | ND | 36043 | 32006 | ND | ND | ND | ND | 10410 | 9525 |
| *Cmtm4* | ND | ND | 74354 | 26442 | ND | ND | ND | ND | 102332 | 215324 |
| *Talpid3* | ND | ND | 48735 | 34775 | ND | ND | ND | ND | 44665 | 30208 |

Data presented represent the total number of CpG sites analyzed at each locus. Note that not all strands analyzed have data from every CpG site due to incomplete sequences or anomalous sites. ND = not determined.
