# Supplementary material for: Methylation is maintained specifically at imprinting control regions but not other DMRs associated with imprinted genes in mice bearing a mutation in the Dnmt1 intrinsically disordered domain
Source: Front Cell Dev Biol. 2023 Aug 4;11:1192789. doi: 10.3389/fcell.2023.1192789 (PMC10436486; doi:10.3389/fcell.2023.1192789)
Supplement: Supplementary file 7 [file Image1.pdf]

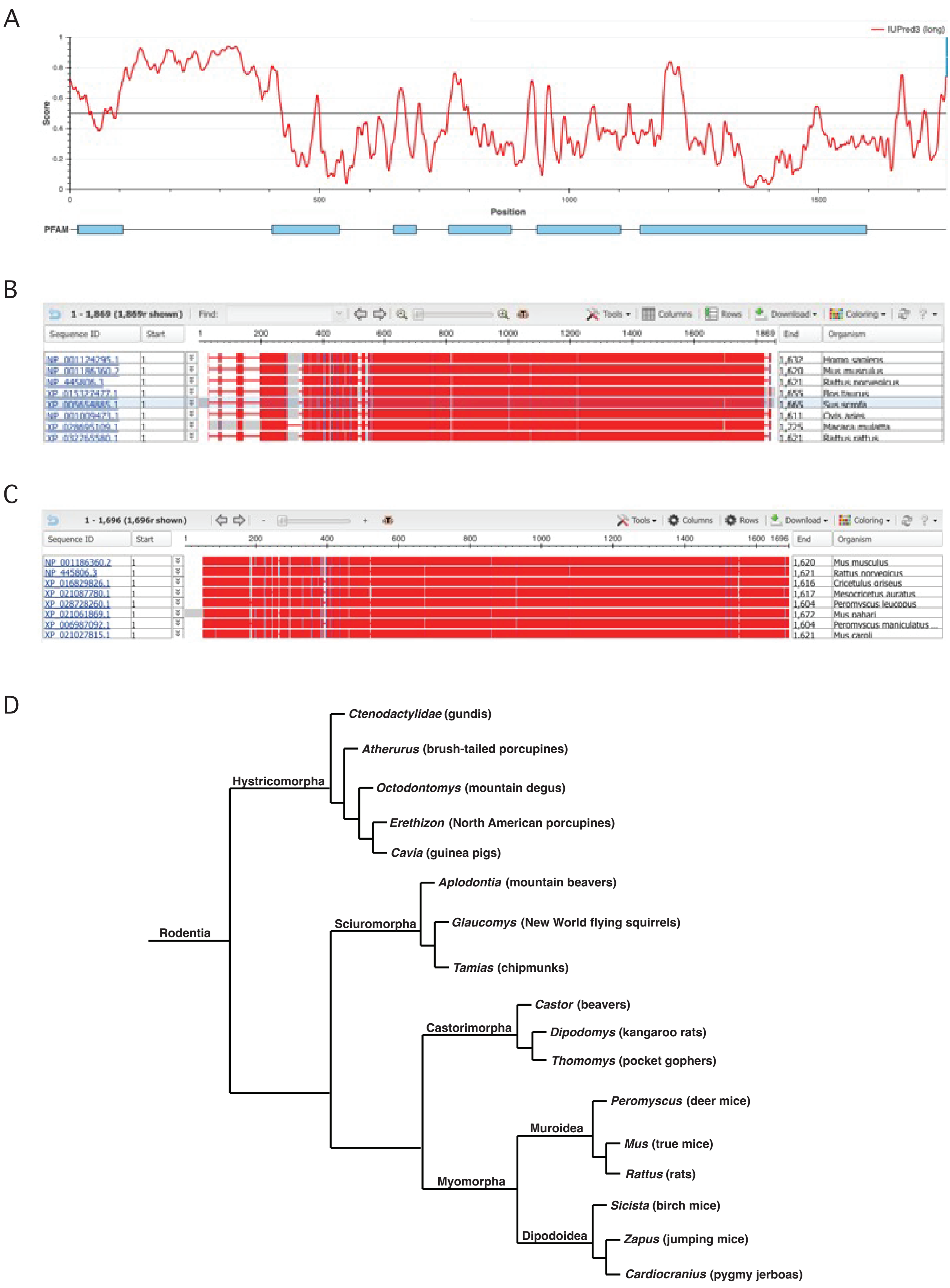

Supplementary Figure S1. The intrinsically disordered domain of Dnmt1 contains a 10 amino acid sequence unique to Mus and Rattus. (**A**) IUPred3 results for Mus musculus Dnmt1 illustrating the intrinsically disordered domain located from amino acids ~100-400 (https://iupred.elte.hu/); disorder score on Y axis. (**B**) Alignment of Dnmt1 sequences from human, mouse, rat (Rattus norvegicus), cattle, pig, sheep, rhesus monkey and rat (Rattus rattus). Sequences are dissimilar in the intrinsically disordered domain across this broad range of species. (**C**) Alignment of Dnmt1 sequences from true mouse (Mus musculus), rat, Chinese hamster, golden hamster, deer mouse (Peromyscus leucopus), true mouse (Mus pahari), deer mouse (Peromyscus maniculatus), true mouse (Mus caroli). Sequences are more similar throughout the intrinsically disordered domain among rodents. (**D**) Classification of rodent families based on (Fabre et al., 2012).
